# The association between part-time and temporary employment and sickness absence: a prospective Swedish twin study

**DOI:** 10.1093/eurpub/cky145

**Published:** 2018-08-02

**Authors:** Björg Helgadóttir, Pia Svedberg, Lisa Mather, Petra Lindfors, Gunnar Bergström, Victoria Blom

**Affiliations:** 1 Division of Insurance Medicine, Department of Clinical Neuroscience, Karolinska Institutet, Stockholm, Sweden; 2 Department of Psychology, Stockholm University, Stockholm, Sweden; 3 Unit of Intervention and Implementation Research for Worker Health, The Institute of Environmental Medicine, Karolinska Institutet, Stockholm, Sweden; 4 Department of Occupational and Public Health Sciences, Centre for Musculoskeletal Research, University of Gävle, Gävle, Sweden; 5 The Swedish School of Sport and Health Sciences, Stockholm, Sweden

## Abstract

**Background:**

Sickness absence (SA) is becoming a major economic problem in many countries. Our aim was to investigate whether type of employment, including temporary employment or part-time employment, is associated with SA while controlling for familial factors (genetic and shared environment). Differences between men and women and across employment sectors were explored.

**Methods:**

This is a prospective twin study based on 21 105 twins born in Sweden 1959–85. The participants completed a survey in 2005 with follow-up of SA (≥15 days), using register data, until end of 2013. The data were analyzed with logistic regression, with results presented as odds ratios (OR) with 95% confidence intervals (CI).

**Results:**

Temporary employment involved higher odds of SA (OR=1.21 95% CI=1.04–1.40) compared to full-time employment. Both part-time workers (OR=0.84 95% CI=0.74–0.95) and the self-employed (OR=0.77 95%CI=0.62–0.94) had lower odds of SA. Stratifying by sex showed lower odds for part-timers (OR=0.82 95% CI=0.73–0.94) and self-employed women (OR=0.65 95% CI=0.47–0.90), but higher odds for men in temporary employment (OR=1.33 95% CI=1.03–1.72). Temporary employees in county councils (OR=1.73 95% CI=1.01–2.99) and municipalities (OR=1.41 95% CI=1.02–1.96) had higher odds while part-timers employed in the private sector had lower odds (OR=0.77 95% CI=0.64–0.93). Familial factors did not confound the association between employment type and SA.

**Conclusions:**

Employment type is associated with SA, with temporary employment involving a higher risk compared to permanent full-time employment while both part-time employment and self-employment involved a lower risk. The associations vary between women and men and across sectors.

## Introduction

Sickness absence (SA) is a complex phenomenon with it being a function of a disease or injury and its effect on work capacity, as well as the insurance rules that apply in a country. The rates of SA vary but are generally high in the Nordic countries.^1^ In Sweden, the number of cases of SA has increased during the past few years,^2^ and the absence duration is increasing.^3^ Various risk factors for SA, apart from disease, have been explored and identified, including old age, being a woman, low socioeconomic status, poor self-rated health (SRH) and previous history of SA.^4–6^ Still, few have studied the effect of type of employment.

Temporary employment can be defined as an employee having a contract which is clearly limited in time, with a specific end date. This can be compared to a permanent employment where the contract includes no end date. Research has shown that temporary employment is negatively associated with occupational health and SRH.^7–10^ However, whether temporary employment also increases the risk of SA remains unclear. A study in the Nordic countries found that temporary employment was associated with a higher risk of SA.^11^ Yet, a study from Finland found that temporary employment was associated with a lower risk of SA than permanent employment.^12^ Another European study investigating both health indicators and SA found that temporary employment had detrimental effects on health, but also showed that temporary employed were less likely to be on SA than full-time permanent workers.^13^ Thus, workers with temporary employment seem more reluctant to be on SA, possibly due to fears of being fired.

Also, other types of employment, including part-time employment which involves working <100%, i.e. less than full-time, or self-employment which for instance involves owning a business or freelancing, have rarely been explored in association to SA. A Danish study of hospital employees found that part-time employment was a risk factor for SA among pregnant women.^14^ However, a Swedish study exploring different proportions of part-time employment found that full-time employment, part-time employment exceeding 50% and self-employment was associated with a lower risk of disability pension, while part-time employment of <50% of full-time was associated with a higher risk of disability pension.^15^ Furthermore, differences between women and men may play a role as women are more likely to work part-time^16^ and be on SA.^4^

Besides type of employment, occupational sector may be important. A cross-sectional study from Finland of differences between occupational sectors found no statistically significant differences in SRH between public and private sectors or between contract types. However, strenuous physical work was found more common among men in the private sector but the least common among women in the private sector, even after stratifying by contract type.^17^ With physical working conditions being one of the main factors in explaining differences in SA between occupational groups, this is important.^18^ Moreover, psychosocial working conditions have been shown to influence the association between occupational groups and SA.^19^

In Sweden, the public sector is split into the state, municipalities and county councils with each having different responsibilities. The state includes governmental institutions such as universities and governmental agencies as well as the parliament and the government. County councils carry a responsibility for healthcare systems including hospitals while the municipalities run the educational system (preschools, schools) and elder care. Data from Swedish authorities from 2016 show that 84% of the workforce, including all sectors, hold a permanent contract.^20^ Yet, compared to men, a slightly lower proportion of women in the private sector have a permanent contract (81% vs. 87%), while the opposite holds for those employed by municipalities (85% for women vs. 78% for men). In Sweden, part-time employment (<34 h per week) is more common among those working in healthcare than in all types of industry (36% vs. 22%).^21^ Thus, there is a need to explore both sex and occupational sector in relation to type of employment.

Genetic liability is an interesting individual factor in the study of SA. Studying familial (genetics and shared environmental) factors using twin cohorts has shown that both SA and disability pension are moderately influenced by genetic factors.^22–25^ Moreover, it is possible that familial factors are associated with individuals ability to work full-time or to get a permanent contract. This means that familial factors should be taken into account when studying factors associated with SA.

### Aim

The aim is to study whether type of employment, in terms of part-time or temporary employment, is associated with future SA among women and men, and by occupational sector, when adjusting for confounders including familial factors (genetics and shared environment).

## Methods

### Study population

The source population consisted of 42 582 twins born between 1959 and 1985 of the Swedish Twin project Of Disability pension and SA (STODS). Of these, 25 496 participated in the Study of Twin Adults: Genes and Environment (STAGE) web-based survey conducted by the Swedish Twin Registry in 2005.^26^ In this study, baseline refers to the date when an individual completed the questionnaire. Individuals missing a response date, being on disability pension, having a SA spell (≥15 days) or having an unspecified work status at baseline were excluded (for further details, see Supplementary Appendix figure A1). The final sample included 6313 complete twin pairs; 2509 monozygotic (MZ) pairs, 1757 same-sex dizygotic (DZ) pairs and 1820 opposite-sex pairs and 227 pairs with unknown zygosity. Also, 8479 single twins were included, i.e. they are considered single twins because the twin sibling did not respond to STAGE, or was excluded based on the above criteria. In total, the sample included 21 105 individuals. For details on zygosity determination in the Swedish Twin Registry, see Magnusson et al.^27^

### Outcome and follow-up time

SA data were obtained from the National Social Insurance Agency Micro Data for Analyses of Social insurance database (MiDAS) and linked to each individual using the Swedish ten-digit personal identification number. All individuals in Sweden above the age of 16, with an income from work or unemployment benefits, can receive sickness benefits paid by the Social Insurance Agency when any disease or injury has reduced their work capacity. Employees receive sick pay from their employers during the first 14 days after a qualifying day without benefits (with self-employed usually having more qualifying days). *SA* was operationalized as having at least one incident spell lasting 15 days or longer during follow-up i.e. between the date of STAGE survey response (varying between 1 November 2004 and 21 April 2006) and 31 December 2013. The outcome variable SA was created and no SA spells during follow-up were used as a reference.

### Exposures

*Type of employment* was measured with the question ‘How have you mainly worked during the last three years?’ with the response alternatives: (i) Permanent employee full-time, (ii) Permanent employee part-time, (iii) Temporary employee full-time, (iv) Temporary employee part-time, (v) Self-employed (including part owner), (vi) It has varied; I have worked, studied and/or been unemployed (vii) Not worked at all. Those responding vi or vii were excluded (Supplementary Appendix figure A1). Response alternatives iii and iv were merged as these categories included fewer individuals.

### Covariates

*Occupational sector* was assessed by the question ‘Who has been your main employer during the past three years?’ with the response alternatives: (i) State, (ii) Municipality, (iii) County council, (iv) Private sector, (v) Self-employed, (vi) Other.

*Age* was included as a continuous variable derived by subtracting the date of response to the STAGE questionnaire from the birthdate. *Sex* was dichotomous (women, men). *Marital status* was grouped into married/cohabiting or other. The highest level of *education* was categorized into three groups (i) Primary, (ii) Secondary or vocational and (iii) Higher education.

To place participants in *socioeconomic position* groups, the question ‘what type of profession/job do you have now or when you were last active in the labor market?’ was used. The six resulting categories were based on a socioeconomic classification system developed by Statistics Sweden, ranging from manual employees (two categories) to non-manual employees (three categories) and self-employed (one category).^28^

The Swedish translation^29^ of the Karasek and Theorell^30^ questionnaire was used to assess *Job demands, control* and *support.* Responses were given on a four-point Likert scale, ranging from 1= do not agree to 4= agree entirely. Mean scores were calculated for job demands, control and support and used as continuous variables.

*SRH* was measured in STAGE with the question ‘How would you rate your general health status?’ with response alternatives excellent, good, moderate, fairly poor and poor. As there were few responses in the lowest categories, ‘fairly poor’ and ‘poor’ were collapsed into one category. *Previous SA* was based on MiDAS data (episodes of SA ≥15 days in a row) between 2003 and STAGE response (approximately a two-year period) and dichotomized into yes or no.

### Statistical analyses

Logistic regression analyses were used to estimate odds ratios (ORs) with 95% confidence intervals (CI), to assess the associations between type of employment and SA. The responses ‘do not know/do not want to answer’ were treated as missing values and not included in the analyses. Clustered robust standard error adjustments were made to the analysis to adjust for the non-independence of the twin pairs. When analyzing the whole sample, covariates were entered in three blocks: first sociodemographic factors (age, education, marital status) were entered (Model 1), then job demands, control and support were entered (Model 2) and finally, previous history of SA and SRH were added (Model 3). Furthermore, the analyses were stratified by sex and occupational sector, respectively. Interaction terms between type of employment and sex were entered into the fully adjusted model. Based on this model, men and women within part-time employment, temporary employment etc. were compared. The same logic was applied to models including occupational sector.

Co-twin control (conditional logistic regression) analyses based on discordant MZ and same-sex DZ twin pairs were conducted to adjust for familial (genetics and shared family environment) confounding.^31^ A twin pair was treated as discordant if only one twin of a pair had incident SA during follow-up. In co-twin control analyses, twins in a pair are optimally matched on genetics (MZ 100% and DZ on average 50%) and shared environmental factors (100%) when reared together, and for age and sex. An influence of familial factors is indicated if an association found in the whole sample disappears or changes considerably in the analyses of discordant twin pairs. Co-twin analyses were conducted stratified both by sex (MZ and DZ pairs combined) and by zygosity. All analyses were conducted using STATA IC 12.1.

This study was approved by the Regional Ethical Review Board in Stockholm, Sweden (Ref. No. 2007/524-31; 2010/1346-32/2).

## Results

Table 1 provides descriptive statistics for all study variables in the full sample. Of the 7258 participants on SA, the majority were on full-time SA (67.6%, *n* = 4905), while 4.9% (*n* = 357) were on <50% SA and 22.3% (1618) were on 50–75% SA (data not shown).

**Table 1:** Descriptive statistics of the 21 105 Swedish twin individuals included in the study population by sick-leave status during follow-up

	Total		No sick-leave		Sick-leave	
Covariates	*n*	(%)	*n*	(%)	*n*	(%)
**Occupational sector past three years** (*N*=15 699)						
State	1480	(9.43)	1007	(9.96)	473	(8.46)
Municipality	3162	(20.14)	1716	(16.97)	1446	(25.88)
County council	1063	(6.77)	605	(5.98)	458	(8.20)
Private sector	8554	(54.49)	5788	(57.24)	2766	(49.50)
Self-employed	1024	(6.52)	729	(7.21)	295	(5.28)
Other	416	(2.65)	266	(2.63)	150	(2.68)
**Type of employment** (*N*=15 745)						
Employed full-time	11 190	(71.07)	7371	(72.70)	3819	(68.12)
Employed part-time	1986	(12.61)	1156	(11.40)	830	(14.81)
Temporary employment	1613	(10.24)	939	(9.26)	674	(12.02)
Self-employed	956	(6.07)	673	(6.64)	283	(5.05)
**Sex** (*N*=21 105)						
Men	9948	(47.14)	7481	(54.03)	2467	(33.99)
Women	11 157	(52.86)	6366	(45.97)	4791	(66.01)
**Age** [mean (SD), range 19–47] (*N*=21 105)	33.4	(7.67)	33.1	(7.75)	34.0	(7.49)
**Education** (*N*=19 430)						
Primary	1048	(5.39)	549	(4.27)	499	(7.58)
Secondary/Vocational	9221	(47.46)	5835	(45.42)	3386	(51.43)
Higher education	9161	(47.15)	6462	(50.30)	2699	(40.99)
**Marital status** (*N*=20 521)						
Married/cohabiting	13 715	(66.83)	8774	(65.23)	4941	(69.88)
Other	6806	(33.17)	4676	(34.77)	2130	(30.12)
**Job demands** [mean (SD), 1–4] (*N*=14 259)	2.5	(0.57)	2.5	(0.56)	2.4	(0.58)
**Control** [mean (SD), 1–4] (*N* =14 482)	1.9	(0.55)	1.9	(0.54)	2.0	(0.56)
**Support** [mean (SD), 1–4] (*N*=13 747)	1.6	(0.48)	1.6	(0.46)	1.7	(0.50)
**Socioeconomic position** (*N*=14 975)						
Manual employees in goods production	2424	(16.19)	1515	(15.65)	909	(17.17)
Manual employees in service production	3832	(25.59)	2188	(22.60)	1644	(31.06)
Non-manual employees lower level	2142	(14.30)	1387	(14.33)	755	(14.26)
Non-manual employees. intermediate level	3848	(25.70)	2554	(26.38)	1294	(24.45)
Non-manual higher level	2420	(16.16)	1799	(18.58)	621	(11.73)
Self-employed	309	(2.06)	239	(2.47)	70	(1.32)
**Previous sickness absence** (*N*=21 105)						
No	17 812	(84.40)	12 495	(90.24)	5317	(73.26)
Yes	3293	(15.60)	1352	(9.76)	1941	(26.74)
**Self-rated health** (*N*=20 255)						
Excellent	6916	(34.14)	4898	(36.94)	2018	(28.85)
Good	9998	(49.36)	6507	(49.08)	3491	(49.90)
Moderate	2943	(14.53)	1664	(12.55)	1279	(18.28)
Fairly poor/poor	398	(1.96)	190	(1.43)	208	(2.97)

Part-time workers had lower odds of being on SA (OR = 0.84 95% CI = 0.74–0.95), even after adjusting for possible confounding factors, compared to full-time workers (Table 2). After stratifying by sex, this held for women only (OR= 0.82 95% CI = 0.73–0.94), while no significant effect emerged for men. The self-employed also had a lower risk, with this relationship being fairly consistent from the crude model to the fully adjusted model. Stratification by sex showed a significantly lower risk for self-employed women only (OR = 0.65, 95% CI = 0.47–0.90); this was somewhat stronger compared to that of analyzing both men and women (OR = 0.77, 95% CI = 0.62–0.94). Finally, temporary workers had higher odds of SA; this was somewhat reduced after adjustment, from an OR of 1.39 (95% CI = 1.24–1.54) in the crude model to 1.21 (95% CI = 1.04–1.40) in the fully adjusted model. The higher odds in the fully adjusted model were significant for men (OR = 1.33 95% CI = 1.03–1.72) but not for women (OR = 1.10 95% CI = 0.93–1.32). Interaction analyses showed that women had a higher risk of SA compared to men for full-timers (*P* < 0.01), part-timers (*P* = 0.03), temporary workers (*P* < 0.01) and the self-employed (*P* = 0.02) (data not shown).

**Table 2:** Associations between type of employment and sickness absence stratified by sex, odd ratios (OR) with 95% confidence intervals (CI)

	Crude		Adjusted 1		Adjusted 2		Adjusted 3	
	OR	(95% CI)	OR	(95% CI)	OR	(95% CI)	OR	(95% CI)
**All**								
Employed full-time	ref							
Employed part-time	1.39	(1.26–1.53)	0.89	(0.80–0.99)	0.88	(0.78–0.99)	0.84	(0.74–0.95)
Temporary employment	1.39	(1.24–1.54)	1.18	(1.05–1.33)	1.19	(1.03–1.37)	1.21	(1.04–1.40)
Self-employed	0.81	(0.70–0.94)	0.81	(0.69–0.95)	0.80	(0.66–0.98)	0.77	(0.62–0.94)
**Women**								
Employed full-time	ref							
Employed part-time	0.94	(0.85–1.05)	0.86	(0.77–0.96)	0.86	(0.76–0.98)	0.82	(0.73–0.94)
Temporary employment	1.18	(1.03–1.35)	1.07	(0.92–1.24)	1.07	(0.90–1.27)	1.10	(0.93–1.32)
Self-employed	0.68	(0.54–0.85)	0.65	(0.51–0.82)	0.69	(0.50–0.95)	0.65	(0.47–0.90)
**Men**								
Employed full-time	ref							
Employed part-time	1.16	(0.82–1.62)	1.22	(0.84–1.77)	1.30	(0.87–1.94)	1.26	(0.85–1.88)
Temporary employment	1.21	(1.00–1.46)	1.35	(1.10–1.67)	1.37	(1.08–1.75)	1.33	(1.03–1.72)
Self-employed	1.03	(0.85–1.25)	0.96	(0.78–1.18)	0.89	(0.69–1.15)	0.85	(0.66–1.10)

Note: Adjusted 1: Sex, age, socioeconomic position, marital status.

Adjusted 2: Sex, age, socioeconomic position, marital status, job demands, control, support.

Adjusted 3: Sex, age, socioeconomic position, marital status, job demands, control, support, self-rated health, previous sickness absence.

Table 3 shows the associations between type of employment and SA stratified by occupational sector. Part-time workers had a lower risk of SA when working in the private sector (OR = 0.77 95% CI = 0.64–0.93). However, temporary employment was associated with increased odds of SA for those employed by municipalities (OR = 1.41 95% CI = 1.02–1.96) and county councils (OR = 1.73 95% CI = 1.01–2.99). The interaction term between occupational sector and type of employment was non-significant overall when added to the model. However, results from the interaction model showed that part-timers in the private sector had lower odds compared to part-timers employed by the state (*P* = 0.01) and municipalities (*P* = 0.01). Moreover, temporary employment in municipalities (*P* = 0.01) and county councils (*P* = 0.02) had higher odds than those employed by the state, while temporary workers in the private sector had lower odds than those employed in municipalities (*P* < 0.01) and county councils (*P* = 0.02). Additionally, full-timers in the private sector had lower odds compared to full-timers in municipalities (*P* < 0.01) and county councils (*P* = 0.04) and full-timers in municipalities had lower odds of SA than those employed by the state (*P* = 0.03).

**Table 3:** Associations between type of employment and sickness absence stratified by occupational sector, odd ratios (OR) with 95% confidence intervals (CI)^a^

	Crude		Adjusted 1		Adjusted 2		Adjusted 3	
Type of employment	OR	(95% CI)	OR	(95% CI)	OR	(95% CI)	OR	(95% CI)
**State**								
Employed full-time	ref							
Employed part-time	1.80	(1.18–2.74)	1.15	(0.73–1.83)	1.31	(0.80–2.16)	1.39	(0.84–2.30)
Temporary employment	0.84	(0.58–1.21)	0.81	(0.54–1.21)	0.72	(0.44–1.19)	0.87	(0.52–1.46)
**Municipality**								
Employed full-time	ref							
Employed part-time	1.13	(0.96–1.34)	0.85	(0.71–1.03)	0.87	(0.71–1.06)	0.85	(0.69–1.04)
Temporary employment	1.38	(1.09–1.76)	1.33	(1.02–1.74)	1.36	(0.99–1.87)	1.41	(1.02–1.96)
**County council**								
Employed full-time	ref							
Employed full-time	1.15	(0.84–1.57)	0.98	(0.70–1.38)	0.98	(0.69–1.40)	0.89	(0.61–1.30)
Temporary employment	1.36	(0.88–2.12)	1.28	(0.79–2.07)	1.64	(0.96–2.79)	1.73	(1.01–2.99)
**Private**								
Employed full-time	ref							
Employed part-time	1.24	(1.07–1.44)	0.83	(0.70–0.98)	0.80	(0.67–0.96)	0.77	(0.64–0.93)
Temporary employment	1.29	(1.06–1.57)	1.13	(0.91–1.40)	1.13	(0.87–1.47)	1.12	(0.85–1.47)
**Self-employed**								
Employed full-time	ref							
Employed part-time	1.40	(0.34–5.72)	0.75	(0.13–4.32)	0.74	(0.10–5.76)	0.34	(0.02–4.78)
Temporary employment	1.23	(0.31–4.88)	1.13	(0.30–4.28)	0.89	(0.17–4.72)	1.24	(0.16–9.81)

Note: Adjusted 1: sex, age, socioeconomic position, marital status.

Adjusted 2: sex, age, socioeconomic position, marital status, job demands, control, support.

Adjusted 3: sex, age, socioeconomic position, marital status, job demands, control, support, SRH, previous sickness absence.

^a^The category of self-employed on the type of employment variable is not included in these analyses.

Table 4 compares results from the full sample adjusted for age and sex, with results from the co-twin model adjusting for familial confounding. The comparison of the adjusted Model 1 and the co-twin model (both MZ and DZ) shows similar results. This suggests that the associations are not confounded by familial factors.

**Table 4:** Discordant twin pair analyses

	Adjusted 1		Co-twin all (MZ+DZ)	
Type of employment	OR	(95% CI)	OR	(95% CI)
Employed full-time	ref			
Employed full-time	0.97	(0.87–1.07)	0.99	(0.74–1.31)
Temporary employment	1.26	(1.13–1.42)	1.34	(0.94–1.92)
Self-employed	0.84	(0.72–0.98)	0.86	(0.55–1.33)

Note: Adjusted 1=whole sample adjusted for age and sex.

Moreover, we did a sensitivity analysis excluding individuals who had been on SA during an approximated two years before the baseline. These results were comparable to that of the main analysis (Supplementary Appendix tables A1–A3).

## Discussion

The present results showed that temporary employment was associated with an increased risk of SA, while both part-time employment and self-employment were associated with a lower risk when compared to full-time employment. Stratification by sex revealed slightly different patterns, with part-time and self-employment being associated with a significantly lower risk among women, while temporary employment was associated with a higher risk among men. Regarding sectors, there was an increased risk of SA for temporary workers employed by county councils and municipalities while part-timers had a lower risk when working in the private sector. These results do not seem to be explained by familial factors, i.e. genetics and shared environment.

The findings showing an increased risk of SA among individuals with temporary employment follows previous research,^11^ and in particular studies including health as an outcome.^7–10^ This can perhaps be explained by the greater psychosocial stress that comes with an insecure employment. Over time, this stress may yield subjective health complaints. This was the case in a study comparing temporary and permanent hotel employees who were doing similar work within the same hotels but with the temporary workers reporting more health complaints than the permanently employed.^32^ This difference was explained by the poorer working conditions, including less control of hours worked, working long hours and a high work intensity.^32^ However, some studies report the opposite.^12^^,^^13^ For example, Virtanen et al.^12^ found that temporary workers had a lower risk of SA of more than three days and a lower risk of poor SRH. The definition of SA (≥3 days vs. ≥15 days) may explain the different results. Moreover, labor market conditions, rules and regulations regarding SA may vary across time periods (i.e. 1998–99 vs. 2004–13) and countries (i.e. Finland vs. Sweden).

The finding that part-time employment is associated with a lower risk of SA needs further exploration. Being part-time employed involves working fewer hours per week, and with more time for non-work activities, including recovery from work, this may involve having a lower risk of being sick during work-hours. Moreover, it is perhaps less stressful to work part-time, due to a lower workload and better work-life balance, which may, in turn, be associated with better health. However, some part-timers may be former full-timers now working part-time due to health issues as fewer hours at work would make it easier for them to cope with their condition. We tried to address this issue by exploring whether excluding individuals with a history of SA during the past two years but observed no differences. However, it is still possible that any change from full-time to part-time occurred earlier or that SA fails to fully capture poor health conditions associated with such a change. Yet, with part-time being associated with lower risks for women, this should mainly be an issue for men. Women working part-time had a lower risk of SA than women working full-time, and this effect was stronger than in men. This may be due to the fact that women typically carry a double workload. Specifically, working women in Sweden—as elsewhere—are more often responsible for household work and childcare and spend more time on these duties than their men partners.^33^^,^^34^ This would make part-time employment a more attractive option, as a strategy to reduce the workload that results from combining full-time work with unpaid work at home.

A previous study investigating differences between private and public sectors found no differences in health-related outcomes with findings being similar for permanent and temporary employees.^17^ However, this study did not differentiate between levels of the public sector. We found that part-timers had a lower risk than full-timers among those working in the private sector. Additionally, the risk was lower when compared to individuals working part-time for the state and municipalities. Moreover, temporary workers in the private sector had lower odds of SA than temporary workers employed by municipalities and the county council, with similar results for those working full-time. Thus, something in the private sector seems associated with less SA among these employees than in their counterparts employed by the public sector. Perhaps a higher pressure to produce and be efficient, which is often considered a characteristic of the private sector, may be coupled with employees fearing to lose their job or miss out on a promotion and thus striving to avoid SAs. This pressure may also be associated with changing sectors or jobs to reduce pressure. However, an in-depth analysis of employees of the private sector is needed to understand this pattern of associations.

Self-employment has previously been shown to involve a lower risk of SA^35^ and disability pension,^15^ and our findings align with these results. However, the mechanisms behind this association are unclear. This may relate to real health benefits of self-employment. But the self-employed may also stay out of any SA, particularly if they feel that their business or employees depend on them being present at work. With the potentially large variations among the self-employed, both explanations may apply.

### Strengths and limitations

An obvious strength of this study involves the large sample size. Also, SA data came from registers which ensured complete coverage of this outcome. Furthermore, we had detailed information regarding type of employment and employment sectors, which is seldom available, especially within one dataset. Using a cohort of twins allowed ruling out familial factors as possible confounders in the association between type of employment and SA.

There are issues relating to the data, which hinder conclusions regarding causality. Although this study used a longitudinal design, reverse causation may have influenced the results. For instance, some part-timers may have worked full-time in the past but health problems may have resulted in them changing to part-time instead. We tried to address this by excluding individuals with a history of SA during the past two years, but found no differences. However, it is possible that any change from full-time to part-time occurred earlier or that SA data fail to fully capture health conditions associated with part-time work. Longitudinal data on SRH could perhaps have been used to correct for any bias. Unfortunately, we had no such longitudinal data. Still, using a twin design allowed adjusting for more confounding than is typically the case, thus making our results more valid.

Other limitations include not having enough power to differentiate between different diagnoses for SA, which would obviously add knowledge. With some missing on the questionnaire data, unmeasured confounding and selection bias may be a problem. Selection bias may have yielded an underestimation of the effects if individuals who responded were healthier than the non-responders. Importantly, the part-timers and temporary workers in this sample were more likely to be manual service workers. This may have influenced the results despite us controlling for socioeconomic position to reduce the impact of this confounder.

## Conclusion

To conclude, this study showed that temporary employment was associated with a higher risk of SA while both part-time employment and self-employment seemed to be associated with a lower risk than permanent full-time employment. These associations varied between women and men and across sectors. There was no confounding by familial factors in the associations between type of employment and SA. This suggests that the association is explained by environmental influences outside the shared environment.

## Supplementary data

Supplementary data are available at *EURPUB* online.

## Funding

This study was financially supported by AFA Insurance (140246). STODS data collection was supported by the Swedish Research Council (521-2008-3054), the Swedish Research Council for Health, Working Life and Welfare (2007-0830) and the Swedish Society of Medicine. We acknowledge The Swedish Twin Registry for access to data. The Swedish Twin Registry is managed by Karolinska Institutet and receives funding through the Swedish Research Council under the grant no 2017-00641. STAGE was supported by the National Institute of Health, USA (grants DK 066134 and CA 085739).

*Conflicts of interest*: none declared.

**Key points**

- The temporary employed had a higher risk of SA than permanently employed full-timers.
- Part-time employees and the self-employed had a lower risk of SA than permanently employed full-timers.
- The association between employment type and SA was partly dependent on sex and employment sector.
- Familial factors (genetics and shared environment) did not influence the association.
- Public health policy should acknowledge linkages between the type of employment and SA, which, in clinical practice, includes asking individuals about their employment to understand health differentials.

## Supplementary Material

Supplementary Figure

Supplementary Table
