# Supplementary figures and images for: The association between part-time and temporary employment and sickness absence: a prospective Swedish twin study
Source: Eur J Public Health. 2018 Aug 2;29(1):147–53. doi: 10.1093/eurpub/cky145 (PMC6345142; doi:10.1093/eurpub/cky145)

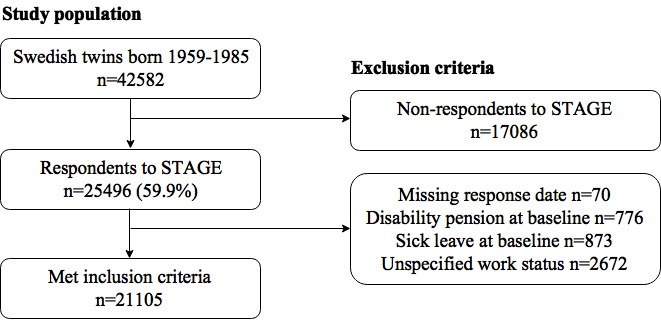

Supplement: Supplementary Figure [file cky145_supplementary_figure.png]
