# Supplementary material for: The association between part-time and temporary employment and sickness absence: a prospective Swedish twin study
Source: Eur J Public Health. 2018 Aug 2;29(1):147–53. doi: 10.1093/eurpub/cky145 (PMC6345142; doi:10.1093/eurpub/cky145)
Supplement: Supplementary Table [file cky145_supplementary_table.docx]

**Table A.1.** Associations between type of employment and sickness absence stratified by sex, Odd Ratios (OR) with 95% Confidence Intervals (CI) excluding those that had been sickness absent during an approximated two years before baseline.

|  | **Crude** | | **Adjusted 1** | | **Adjusted 2** | | **Adjusted 3** | |
| --- | --- | --- | --- | --- | --- | --- | --- | --- |
|  | **OR** | **(95% CI)** | **OR** | **(95% CI)** | **OR** | **(95% CI)** | **OR** | **(95% CI)** |
| **All** |  |  |  |  |  |  |  |  |
| Employed full-time | **ref** | |  |  |  |  |  |  |
| Employed part-time | 1.31 | (1.17-1.47) | 0.85 | (0.75-0.97) | 0.84 | (0.73-0.97) | 0.82 | (0.72-0.95) |
| Temporary employment | 1.36 | (1.21-1.54) | 1.15 | (1.00-1.32) | 1.14 | (0.97-1.34) | 1.15 | (0.98-1.36) |
| Self-employed | 0.79 | (0.67-0.93) | 0.76 | (0.64-0.92) | 0.70 | (0.56-0.89) | 0.69 | (0.55-0.88) |
|  |  |  |  |  |  |  |  |  |
| **Women** |  |  |  |  |  |  |  |  |
| Employed full-time | **ref** | |  |  |  |  |  |  |
| Employed part-time | 0.92 | (0.81-1.04) | 0.82 | (0.72-0.94) | 0.83 | (0.72-0.96) | 0.82 | (0.71-0.94) |
| Temporary employment | 1.15 | (0.99-1.34) | 1.01 | (0.86-1.20) | 1.00 | (0.82-1.22) | 1.02 | (0.84-1.24) |
| Self-employed | 0.65 | (0.50-0.85) | 0.61 | (0.46-0.81) | 0.58 | (0.39-0.86) | 0.57 | (0.38-0.84) |
|  |  |  |  |  |  |  |  |  |
| **Men** |  |  |  |  |  |  |  |  |
| Employed full-time | **ref** | |  |  |  |  |  |  |
| Employed part-time | 1.01 | (0.68-1.49) | 1.07 | (0.70-1.64) | 1.06 | (0.66-1.69) | 1.07 | (0.66-1.71) |
| Temporary employment | 1.22 | (0.99-1.50) | 1.36 | (1.07-1.72) | 1.35 | (1.03-1.78) | 1.36 | (1.02-1.79) |
| Self-employed | 0.96 | (0.78-1.20) | 0.88 | (0.70-1.11) | 0.79 | (0.59-1.05) | 0.77 | (0.58-1.03) |
|  |  |  |  |  |  |  |  |  |
| Adjusted 1: Sex, age, socioeconomic position, marital status.  Adjusted 2: Sex, age, socioeconomic position, marital status, job demands, control, support.  Adjusted 3: Sex, age, socioeconomic position, marital status, job demands, control, support, self-rated health. | | | | | | | | |

**Table A.2**. Associations between type of employment and sickness absence stratified by occupational sector, Odd Ratios (OR) with 95% Confidence Intervals (CI) ) excluding those that had been sickness absent during an approximated two years before baseline.^*^

| **STATE** | **Crude** | | **Adjusted 1** | | **Adjusted 2** | | **Adjusted 3** | |
| --- | --- | --- | --- | --- | --- | --- | --- | --- |
| **Type of employment** | **OR** | **(95% CI)** | **OR** | **(95% CI)** | **OR** | **(95% CI)** | **OR** | **(95% CI)** |
| Employed full-time | **ref** |  |  |  |  |  |  |  |
| Employed part-time | 1.70 | (1.06-2.73) | 1.02 | (0.61-1.70) | 1.17 | (0.67-2.04) | 1.20 | (0.69-2.10) |
| Temporary employment | 0.97 | (0.66-1.43) | 0.93 | (0.60-1.44) | 0.80 | (0.46-1.37) | 0.85 | (0.49-1.47) |
| **MUNICIPALITY** | | | | | | | | |
| **Type of employment** |  |  |  |  |  |  |  |  |
| Employed full-time | **ref** |  |  |  |  |  |  |  |
| Employed part-time | 1.09 | (0.89-1.33) | 0.82 | (0.65-1.02) | 0.82 | (0.65-1.04) | 0.81 | (0.64-1.03) |
| Temporary employment | 1.43 | (1.10-1.87) | 1.33 | (0.98-1.80) | 1.41 | (0.99-2.01) | 1.40 | (0.98-2.00) |
| **COUNTY COUNCIL** | | | | | | | | |
| **Type of employment** |  |  |  |  |  |  |  |  |
| Employed full-time | **ref** |  |  |  |  |  |  |  |
| Employed full-time | 1.24 | (0.86-1.78) | 1.02 | (0.68-1.51) | 0.96 | (0.63-1.46) | 0.94 | (0.62-1.44) |
| Temporary employment | 1.17 | (0.71-1.95) | 1.07 | (0.60-1.89) | 1.35 | (0.72-2.53) | 1.44 | (0.76-2.72) |
| **PRIVATE** | | | | | | | | |
| **Type of employment** |  |  |  |  |  |  |  |  |
| Employed full-time | **ref** |  |  |  |  |  |  |  |
| Employed part-time | 1.14 | (0.96-1.36) | 0.79 | (0.65-0.96) | 0.78 | (0.63-0.96) | 0.77 | (0.62-0.95) |
| Temporary employment | 1.33 | (1.07-1.66) | 1.15 | (0.90-1.47) | 1.17 | (0.87-1.57) | 1.19 | (0.88-1.61) |
| **SELF-EMPLOYED** | | | | | | | | |
| **Type of employment** |  |  |  |  |  |  |  |  |
| Employed full-time | **ref** |  |  |  |  |  |  |  |
| Employed part-time | 0.66 | (0.07-5.90) | 0.40 | (0.04-4.18) | 0.25 | (0.02-4.00) | 0.04 | (0.00-0.55) |
| Temporary employment | 1.10 | (0.21-5.75) | 0.86 | (0.18-4.14) | 0.32 | (0.06-1.87) | 0.23 | (0.03-1.75) |
| Adjusted 1: sex, age, socioeconomic position, marital status.  Adjusted 2: sex, age, socioeconomic position, marital status, job demands, control, support.  Adjusted 3: sex, age, socioeconomic position, marital status, job demands, control, support, SRH.  *The category of self-employed on the type of employment variable is not included in these analyses. | | | | | | | | |

**Table A.3**. Discordant twin pair analyses excluding those that had been sickness absent during an approximated two years before baseline.

|  | **Adjusted 1** | | **Co-twin all (MZ+DZ)** | |
| --- | --- | --- | --- | --- |
| **Type of employment** | **OR** | **(95% CI)** | **OR** | **(95% CI)** |
| Employed full-time | **ref** |  |  |  |
| Employed full-time | 0.93 | (0.83-1.05) | 0.81 | (0.56-1.18) |
| Temporary employment | 1.23 | (1.08-1.40) | 1.51 | (0.96-2.38) |
| Self-employed | 0.81 | (0.68-0.96) | 0.74 | (0.43-1.26) |
| Note: Adjusted 1=whole sample adjusted for age and sex | | | | |

**Figure legends (Appendix)**

**Figure A.1.** Study population, inclusion and exclusion criteria. STAGE= the Study of Twin Adults: Genes and Environment.
